# Gut Microbiota Profile and the Impact of Probiotic Supplementation in Competitive Cyclists: A Scoping Review

**DOI:** 10.3390/nu18060991

**Published:** 2026-03-20

**Authors:** Giacomo Belmonte, Marco Gervasi, Deborah Agostini, Sabrina Donati Zeppa, Eugenio Formiglio, Irene Rosa Di Mitri, Eneko Fernández-Peña, Alessia Bartolacci, Vilberto Stocchi, Antonio Paoli, Antonino Bianco, Antonino Patti

**Affiliations:** 1 Sport and Exercise Sciences Research Unit, Department of Psychology, Educational Science and Human Movement, University of Palermo, 90144 Palermo, Italy; giacomo.belmonte@unipa.it (G.B.); irenerosa.dimitri@you.unipa.it (I.R.D.M.); antonino.bianco@unipa.it (A.B.); 2 Department of Biomolecular Sciences, Division of Exercise and Health Sciences, University of Urbino Carlo Bo, 61029 Urbino, Italy; marco.gervasi@uniurb.it (M.G.); deborah.agostini@uniurb.it (D.A.); sabrina.zeppa@uniurb.it (S.D.Z.); e.formiglio@campus.uniurb.it (E.F.); a.bartolacci2@campus.uniurb.it (A.B.); 3 Department of Human Science for Promotion of Quality of Life, University San Raffaele, 00166 Rome, Italy; vilberto.stocchi@uniroma5.it; 4 Department of Physical Education and Sport, University of the Basque Country UPV/EHU, 01007 Vitoria-Gasteiz, Spain; eneko.fernandezp@ehu.eus; 5 Department of Biomedical Sciences, University of Padua, 35131 Padua, Italy

**Keywords:** gut microbiota, probiotics, competitive cycling, performance

## Abstract

**Background/Objectives**: The recent discovery of the importance of gut microbiota has enhanced our understanding of several issues related to energy metabolism, immune systems, and post-exercise recovery, which could have an impact on sports performance. Probiotics are used as sports supplements and have recently been proposed to be effective in reducing the incidence of gastrointestinal and respiratory infections during training and competition. This scoping review aimed to evaluate the gut microbiota composition of competitive cyclists and investigate the effect of probiotic administration in this sports population. **Methods**: A literature review was conducted using the following databases: PubMed/Medline, Web of Science, and Scopus, and all studies until 1 November 2025 were considered. After dual-reviewer screening, data were charted to identify the composition of gut microbiota and the effects of probiotics on these types of athletes. **Results**: After all the study identification phases, eleven studies were selected. Seven studies evaluated the composition of the gut microbiota, while four randomized controlled trials evaluated probiotic intake. The results indicate an abundance of Prevotella distinct for this type of athlete, which could facilitate the metabolism of glucose and short-chain fatty acids. Among the four main areas of improvement identified in relation to probiotics, a 16-week multi-strain supplementation protocol showed improved aerobic performance and exertion rate in amateur cyclists. **Conclusions**: Despite the limited number of studies, certain microbiota traits could be identified in competitive cyclists, which may correspond to their high metabolic rate. Although further strain standardized studies are needed on professional cyclists, the data could indicate that certain probiotic supplementation may be an effective addition for competitive cyclists.

## 1. Introduction

The gut microbiota is a collection of microorganisms that live in the human body, including bacteria, archaea, viruses, and eukaryotic microbes [1,2]. The main bacterial groups within the body can include *Firmicutes*, *Bacteroidota*, *Cyanobacteria*, *Pseudomonadota*, *Fusobacteria*, *Actinomycetota*, and *Verrucomicrobiota* [3]. The influence of gut microbiota on various key bodily functions, from the immune system to metabolism, has been demonstrated [4,5]. As a result, interest has grown among professionals and researchers, acknowledging how essential it is to the human digestive system [6]. Recent studies have shown that energy metabolism, inflammation, and recovery in athletes are affected by microbiota [7,8]. Competitive cycling imposes intense physical demands on the body, characterized by lengthy, high-intensity training sessions and competitions. Professional road cycling serves as a primary model for these dynamics, given the extreme endurance demands of the sport. Elite cyclists cover a total of 30,000–35,000 km per year, divided between training and racing around the world, from February to October, exposing cyclists to a wide range of weather conditions [9,10]. In addition, professional road cyclists compete in the Grand Tours, which consist of three consecutive weeks of racing (approximately 100 h of competition), placing extreme physiological and metabolic demands on the body [10]. In this context, a particular diet and, more importantly, rigorous training can affect the levels of microbiota, which in turn affect metabolism and athletic performance [7,11]. The microbiome can aid in maintaining the homeostasis of the intestinal lining, which also affects the brain–gut axis [6,12]. Consequently, they can assist in controlling mental fatigue by reducing neuroinflammation, making them especially valuable in endurance sports that are long-lasting and at high intensity [6,8]. Additionally, it is recognized that exercise intensity can influence the composition of the gut microbiota, particularly its diversity [6]. Evidence suggests that athletes exhibit significantly greater intestinal mycobiome pathways than sedentary counterparts, reflecting a physiological adaptation driven by rigorous training regimens and specialized dietary patterns [13,14,15]. Indeed, in the study conducted by Barton et al., athletes showed a higher concentration of amino acid biosynthesis and fecal metabolites compared to the non-athletic control group [13]. On the contrary, several studies have reported an inverse correlation between intense exercise and gastrointestinal health, noting a decline in microbial diversity among elite athletes [16,17]. This phenomenon has been observed in some endurance athletes, with a relatively reduced abundance of short-chain fatty acid (SCFA)- and lactic acid-producing bacteria [16]. Indeed, such training could compromise the intestinal barrier and increase intestinal permeability, facilitating the translocation of bacteria from the colon and potentially compromising the stability of the microbiome [18]. At the same time, intrinsic adaptations to intense endurance training, such as decreased blood flow and tissue hypoxia, could lead to changes in the gastrointestinal tract, reducing its diversity and exposing athletes to gastrointestinal discomfort [19]. The large training loads and competition demands associated with cycling disciplines lead to substantial energy expenditure, often difficult to offset through dietary intake, resulting in low energy availability [20]. For this reason, various sports supplements are widely used to carry out proper nutritional planning, increase sports performance, and optimize recovery [21,22,23]. Following a recent study conducted by Garcia-Durán et al., it is possible to analyze the supplements most used in competitive cycling [24]. The authors divided the supplements analyzed into the following categories: sports foods, medical supplements, and ergogenic aids [24]. Most supplements used were sports foods, such as sports bars, sports gels, and sports drinks [24]. Among medical supplements, multivitamins were the most widely used, followed by iron and probiotics [24]. Probiotics are popular supplements used by endurance athletes for multiple reasons [25,26]. Several studies have investigated the positive effects of probiotic exposure on aerobic performance parameters, such as running performance in triathletes with 4 weeks of supplementation [27]. At the same time, recently, a 5-week probiotic intake protocol found significant effects on running performance in marathon runners [28]. While probiotics are known to enhance athletic performance and alleviate gastrointestinal symptoms in various cohorts, their specific efficacy remains under-explored among competitive cyclists [29,30]. Postural gastrointestinal stress associated with prolonged aerodynamic positioning for high-volume training, the extreme physiological demands of Grand Tours, and specific nutritional periodization, often characterized by high carbohydrate availability, potentially exerts unique selective pressure on the microbial ecosystem. Gastrointestinal symptoms can be categorized into upper-tract symptoms, such as a feeling of nausea, vomiting, and chest pain, and lower gastrointestinal tract symptoms, such as diarrhea and flatulence [31]. These symptoms are often associated with endurance sports, with a frequency as high as 90% in marathon runners [32]. Competitive cycling, along with triathlon and running, represents one of the sports most affected by these types of symptoms [33,34]. However, to the best of our knowledge, no review has ever analyzed the effects of probiotic supplementation exclusively on competitive cyclists. Furthermore, no review has attempted to find common traits in the gut microbiota composition of these competitive cyclists [35,36]. A scoping review was conducted to outline the breadth of available evidence, given the heterogeneous nature of existing studies on sports nutrition and cycling. Therefore, this study aimed to achieve two objectives: (a) to identify common aspects of gut microbiota composition in competitive cyclists, and (b) to evaluate the effects of probiotic-based sports supplementation in competitive cycling.

## 2. Materials and Methods

This scoping review was performed in accordance with the guidelines outlined in the Preferred Reporting Items for Systematic Reviews and Meta-Analyses Extension for Scoping Reviews (PRISMA-ScR) [37]. The research question was recorded in the “Open Science Framework” (Registration DOI: 10.17605/OSF.IO/VWKH8 registered on 4 March 2026).

### 2.1. Data Sources and Search Strategy

The literature search was conducted on 12 November 2025. Two separate search processes were conducted using the databases PubMed/Medline, Web of Science (WOS), and Scopus (Elsevier). We employed the following search string: (“gut microbiota” OR “microbiome”) AND (“competitive cyclists” OR “elite cyclists” OR “professional cyclists” OR “amateur cyclists”). Subsequently (“probiotic supplementation” OR “probiotics”) AND (“cycling” OR “competitive cycling” OR “elite cycling” OR “professional cycling” OR “amateur cycling”). The full search strategy was also reported for greater transparency of the literature review (Supplementary Material—Table S1).

### 2.2. Eligibility Criteria

The Population, Concept, and Context (PCC) criteria were used.

Population of interest: Well-trained competitive cyclists: amateurs, under-23s, elite, professionals from road cycling, mountain biking, or track cycling.

Concept of interest: Microbiota composition AND/OR probiotic supplementation.

Context of interest: Sports performance and gastrointestinal health.

Inclusion criteria: All articles published and written in English until 1 November 2025 that analyzed the composition of gut microbiota in competitive cyclists were considered. To ensure a comprehensive overview of the evidence, this review included studies of heterogeneous designs, including randomized controlled trials (RCTs), crossover studies, observational studies, and cross-sectional analyses. The articles that analyzed non-competitive cyclists and other sports together were excluded. All results that were analyzed by the included articles were considered. Studies involving mixed-sport cohorts were only included if data pertaining to the target population were reported independently.

Exclusion criteria: Studies involving unspecified endurance athletes were excluded during the screening of titles and abstracts. Studies involving recreational cyclists were also excluded at this stage, as their training volume and dietary characteristics could have influenced considerations regarding the composition of the microbiota. Studies not written in English, conference abstracts, or those that did not specifically include a group of competitive cyclists explicitly described were excluded.

### 2.3. Study Selection Process and Data Extraction

All articles extracted from the databases were uploaded into EndNote 20, a title/abstract screening software program. At first, two researchers worked separately to analyze the results, eliminating duplicates and filtering results by title and abstract. Subsequently, an analysis of the remaining studies was carried out through the full texts. In case of disagreement, the opinion of a third researcher was sought. Relevant data were extracted from the included studies: primary author(s), publication year, participant characteristics, any variables related to microbiota composition or the effects of probiotic intake, and major findings. Percentages of bacteria or changes in outcomes were also extracted. In line with scoping review methodology (PRISMA-ScR), a formal risk-of-bias appraisal was not performed, as the primary objective was to map the extent and nature of the available evidence [37].

## 3. Results

### 3.1. Studies’ Identification

**Figure 1:**
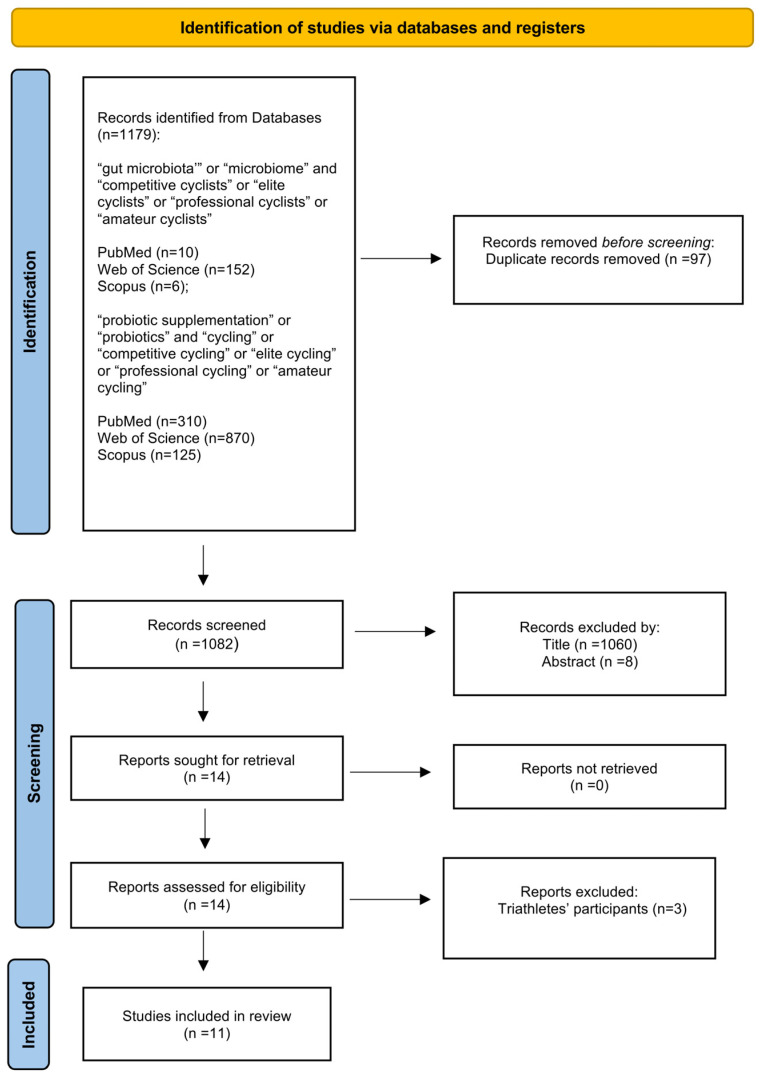
Prisma Flow diagram study selection and eligibility screening.

A total of 1179 articles were initially identified. After the duplicate removal phase, an analysis was conducted on titles and abstracts of the remaining 1082 articles. Subsequently, 14 studies were identified. After a full-text review, 3 studies were excluded because they did not meet the inclusion criteria of the review, and a total of 11 studies were finally included. The three studies were excluded because they included triathletes. Exposure to different training loads involving a combination of multiple sports (cycling, swimming, and running) could alter the composition of the gut microbiota compared to competitive cycling alone. Figure 1 provides a detailed PRISMA flow diagram outlining the study identification, screening, and inclusion process [38].

### 3.2. Study Characteristics

This review analyzed a total of 363 participants across eleven original studies. Seven studies were identified regarding the composition of the gut microbiota in competitive cyclists [39,40,41,42,43,44,45]. On the other hand, four randomized controlled trials evaluated the impact of probiotic supplementation [46,47,48,49]. The studies included professional cyclists (n = 4) [39,41,42,45], elite cyclists (n = 2) [43,48], and trained amateur cyclists (n = 5) [40,44,46,47,49]. The classification of competitive cyclists (professional, well-trained/elite, amateur/trained) was carried out in accordance with the classification proposed by Sitko et al. for studies in which this was not specified [50]. Six studies were conducted through a cross-sectional design [39,40,41,43,44,45], and five were longitudinal [42,46,47,48,49]. Six studies evaluated only male cyclists [40,42,44,47,48,49]; four studies evaluated both male and female cyclists [39,41,45,46], and one evaluated only female cyclists [43].

### 3.3. Gut Microbiota Assessment

Methodological heterogeneity was observed in microbiota assessment. Two studies were performed using shotgun metagenomics and metatranscriptomics [39,45]; five studies were fecal analysis and microbial abundance quantification [40,41,42,43,44]. More specifically, four studies utilized 16S rRNA gene sequencing [41,42,43,44]; one study used Quantitative Real-Time PCR (qPCR) [40]. The detailed characteristics and main findings of the included studies regarding gut microbiota composition are summarized in Table 1.

### 3.4. Probiotics Supplementation

In terms of probiotic strains, only one study used a single strain [46], while the remaining three studies involved participants consuming a multi-strain probiotic [47,48,49]. Probiotic protocols exhibited variability in dosage, ranging from low to high doses. One study used a daily dosage of 1 × 10^11^ [49]; two studies 1 × 10^10^ [47,48]; and one study 1 × 10^9^ [46]. All probiotic trials were placebo-controlled. The outcomes analyzed were physiological [48,49], inflammatory [46,48,49], gastrointestinal or respiratory illness and discomfort [46,47,48], metabolic and oxidation [47], permeability [49] and composition of microbiota [46]. During the study protocols, the training load analysis was measured using online diaries (n = 2) [46,49]; power meters (n = 1) [48]; and only one study used a standardized training [47]. The detailed characteristics and main findings of the included studies regarding probiotic supplementation are summarized in Table 2.

## 4. Discussion

This scoping review had dual objectives: to summarize the common traits of the gut microbiota in competitive cyclists and to explore probiotics’ positive effects on competitive cycling. The rapid and recent increase in interest and knowledge around the probiotics’ intake and the composition of gut microbiota in endurance athletes led to numerous studies in the scientific literature [25,26]. However, the included studies exhibit heterogeneity in terms of probiotic strains, dosages, and exposure durations, as well as the parameters evaluated. On the one hand, this makes it possible to compare the effects of different lengths of probiotic intake in the population examined; on the other hand, it represents a limitation of the study. Furthermore, to the best of the authors’ knowledge, this is the first review to analyze the gut microbiota composition exclusively in competitive cyclists, rather than grouping them with other endurance athletes or general sporting cohorts.

### 4.1. Gut Microbiota Composition

This review outlines substantial differences in the composition of the cyclists’ microbiota, classified into distinct categories, despite their competitive nature. In the study conducted by Petersen et al., a comparison was made between the microbiota of competitive cyclists of different categories: from amateurs to professional cyclists [39]. Regardless of the cycling level analyzed, cyclists found a high concentration of *Prevotella*, a high presence of *Bacteroides*, or a combination of different species, including *Eubacterium*, *Ruminococcus*, *Akkermansia*, and *Bacteroides* [39]. *Prevotella* abundance appears to be influenced by weekly training volume, and relevant to carbohydrate and branched-chain amino acid metabolism [39]. Moreover, professional cyclists showed higher amounts of *Methanobrevibacter smithii* than amateur cyclists in lower categories. Therefore, this type of bacteria might be a predominant part of the professional cycling community [39]. On the other hand, in the study conducted by Wiącek et al., only amateur cyclists were studied by the assessment of total gut bacteria and fecal pH before and after the racing season and an increase in carbohydrate consumption [40]. The authors concluded that there was no difference in the parameters analyzed, even after increasing carbohydrate consumption during the competitive season [40]. These findings might be in accordance with Aya et al., who studied the fecal samples of Colombian competitive cyclists by analyzing their bacterial composition [41]. The results revealed a higher percentage of Archaea in the competitive cycling group than in the weightlifting group, indicating an adaptation to endurance sport. [41]. In another study, Aya et al. combined metagenomic, metabolomic, and lipidomic analyses with previous findings [45]. The study indicates that there are particular microbial signatures that can be differentiated between the two athletic groups. Specifically, cyclists always have a higher relative abundance of *Prevotella*, a genus known for its increased carbohydrate metabolism and short-chain fatty acid production, which could help improve endurance performance [45]. However, these differences in microbes, although significant, are still small when considering the overall species present and fecal functional profiles, which are quite similar in both groups [45]. Although there is no significant divergence in the microbiome and fecal metabolome, it is clear that the most distinct differences are found in the systemic metabolic responses, which are identified through plasma metabolomics and lipidomics analysis [45]. Instead, in the study conducted by Fernandez-Sanjurjo et al., an assessment of the gut microbiota of professional cyclists during a Grand Tour championship was carried out [42]. This competition consisted of 21 consecutive days of stages, and the assessment of the intestinal microbiota based on fecal samples was done at four different times: one day before the initiation of the first stage, after completion of nine stages, and after completion of the last stage [42]. However, no correlation was found between performance and SCFA concentration. The pre-race consumption of complex carbohydrates was associated with an increase in *Erysipelotrichaceae*, while the in-race consumption of simple carbohydrate supplements was associated with a decrease in *Bifidobacteriaceae* [42]. However, Shalmon et al. analyzed the gut microbiota of competitive endurance cyclists and found it to be comparable to that of runners and non-athletes [44]. Cyclists did not differ significantly from controls in alpha or beta diversity, suggesting overall stability in microbial diversity and composition. However, several taxonomic features were identified to distinguish cyclists [44]. Cyclists also had a lower abundance of Enterobacteriaceae, suggesting a potentially healthier gut environment [44]. Moreover, more refined patterns were observed after stratification for sex, and these included enrichments of Coriobacteriaceae, *Bifidobacterium*, and Pseudomonas in male cyclists, and in female cyclists, enrichments were observed in several SCFA producers, including *Lachnospiraceae*, *Rumino-coccaceae*, *Dialister*, *Phascolarctobacterium*, and most significantly, *Prevotella* [44]. The *Prevotella* and *Lachnospiraceae* abundance was positively correlated with training volume, suggesting that training load could influence the bacterial composition of the cyclist [44]. Correlations with performance were also found in cyclists. Dialister abundance was correlated with lactate concentration and time to exhaustion, and *Prevotella* abundance with weekly training volume [44]. The microbiome of cyclists exhibits small overall differences in community composition, which are driven primarily by specific taxa participating in SCFA production and carbohydrate fermentation [44]. Conversely, the study carried out by Ampe et al. examined the gut microbiota of fourteen elite female World Tour cyclists during a period of reduced training, as compared to thirteen age-matched non-athlete controls [43]. Using 16S rRNA sequencing and fecal SCFA quantification, the researchers observed marked compositional differences despite comparable SCFA levels [43]. At the phylum level, cyclists’ microbiota was dominated by *Bacteroidota* (72.7%), with a lower abundance of *Firmicutes* (22.1%) compared to controls (*Firmicutes* 62.5%, *Bacteroidota* 15.3%), alongside a reduction in *Actinobacteriota* [43]. Alpha diversity was reduced in cyclists, and family-level analysis showed a significant reduction in fiber-fermenting bacteria, including *Lachnospiraceae*, *Ruminococcaceae*, *Peptostreptococcaceae*, *Bacillaceae*, *Erysipelotrichaceae*, *Anaerovoracaceae*, and the *Coprostanoligenes* group; no significant differences were found for families of *Bacteroidota* [43], but Actinobacteriota families such as *Bifidobacteriaceae*, Coriobacteriaceae, and Eggerthellaceae were increased in controls [43]. The nutritional and physiological demands of elite cycling, such as high carbohydrate diets, low fiber diets, and methods for optimizing glycogen availability and preventing gastrointestinal disturbances during competition, are likely represented in these differences [51]. The observed reduction in classical butyrate producers across some studies does not necessarily imply a decrease in total SCFA concentrations. It has been hypothesized that functional adaptations, such as the potential upregulation of fermentation genes in taxa like *Bacteroidota*, might maintain SCFA stability [43]. However, evidence remains limited. These findings highlight significant heterogeneity in the literature: while some cohorts align with low-fiber diet profiles, others diverge from the previously suggested increases in *Prevotella* typically associated with endurance athletes. Furthermore, the evidence suggests that microbial patterns involving *Prevotella*, *Bacteroides*, and SCFA-producers may be modulated by individual factors such as biological sex and competitive level. Specifically, variations in the *Bacteroidota* to *Firmicutes* ratio and the abundance of *Lachnospiraceae* and *Ruminococcaceae* appear more pronounced in elite and female cyclists. While these patterns suggest a potentially specialized microbial signature in response to high-volume training, the substantial confounding from dietary intake and the observational nature of most included studies preclude a definitive characterization of a ‘performance-optimized’ microbiome. Consequently, these findings should be interpreted as a guide to possible biomarkers rather than as confirmed functional adaptations.

### 4.2. Probiotics Effects

This review also summarizes the effects of probiotics on competitive cyclists, exploring the effects of this supplement in this specific target population.

#### 4.2.1. Gastrointestinal and Respiratory Symptoms

Several studies evaluated probiotic supplementation on gastrointestinal and respiratory symptoms in competitive cyclists. In the study conducted by West et al., the authors found that male cyclists reported an increase in the duration and frequency of gastrointestinal symptoms and a reduction in severity following probiotic supplementation [46]. These results appear to increase with training intensity [46]. While probiotic supplementation did not yield significant improvements in upper respiratory tract infection (URTI) symptoms, a meaningful clinical impact was observed regarding lower respiratory tract symptoms [46]. On the other hand, female cyclists reported an increase in respiratory disorders, indicating uncertainty in the results regarding URTI [46]. In agreement with these findings, the study conducted by Schreiber et al. found a low occurrence of gastrointestinal symptoms in the cyclist group that supplemented with probiotics [48].

#### 4.2.2. Intestinal Permeability

Regarding intestinal permeability, there was no significant difference in intestinal permeability, as measured by the ratio of lactulose to rhamnose, according to the results of Pugh et al. [47]. Conversely, in the study conducted by Mazur et al., a statistically significant difference in intestinal membrane permeability was observed [49]. To be specific, the group of competitive cyclists who received the probiotics showed a reduction in the level of zonulin [49]. Consequently, it would seem that further studies are needed in this field.

#### 4.2.3. Anti-Inflammatory Role

Several factors were taken into account to assess the anti-inflammatory response of probiotics. West et al. reported that there were no significant alterations in the concentrations of different cytokines, such as IL-1RA, IL-6, IL-8, IL-10, tumor necrosis factor (TNF), and interferon gamma (INF-g) after the intake of probiotics [46]. In line with the above study, the study conducted by Schreiber et al. reported that there were no significant alterations in the concentrations of different inflammatory cytokines such as IL-6, TNFα, and CRP [48]. Contrary to the above studies, in the study conducted by Mazur et al., the concentrations of inflammatory markers during cycling exercise were assessed [49]. There was a significant rise in IgA concentrations, and all other inflammatory cytokines were reduced: TNF-alpha, IL-6, IL-10, and TOS [49]. However, to confirm these results, a larger sample size would be needed, with a single strain dosage rather than multi-strain concentrations.

#### 4.2.4. Cycling Performance

Only three studies have investigated performance levels in cycling with probiotic supplementation [47,48,49]. In the study conducted by Pugh et al., peak oxidation rates of ingested maltodextrin were slightly higher in the Probiotic group than in the Placebo group, along with mean carbohydrate oxidation and a mild decrease in fat oxidation [47]. Nevertheless, four weeks of multi-strain probiotic supplementation did not lead to an improvement in time trial cycling performance of 120 min [47]. Instead, in the study conducted by Schreiber et al., a VO_2_max test and a time-to-fatigue (TTF) test were administered after a 12-week multi-strain probiotic intake of 12 weeks [48]. No significant effects were found in cycling tests. Nevertheless, the rate of perceived exertion (RPE) during TTF was found to be significantly lower, indirectly leading to an improvement in cycling performance [48]. Meanwhile, in the study conducted by Mazur-Kurach et al., a multi-strain probiotic for 16 weeks was administered to competitive cyclists, evaluating Aerobic and Anaerobic tests [49]. For the aerobic test, an incremental test was administered, including maximum oxygen consumption (VO_2_max), exercise time, maximum power achieved, maximum heart rate, and RPE [49]. As regards the anaerobic test, a Wingate test was administered, including the maximum power achieved, the average time to reach maximum power, and the time to maintain the power achieved [49]. In the aerobic test after supplementation, VO_2_max increased significantly, along with an increase in exercise time and a reduction in HR and RPE [49]. As regards the anaerobic test, none of the parameters analyzed showed a statistically significant variation.

### 4.3. Limitations and Future Studies

The studies included have mentioned several limitations that need to be taken into consideration during the interpretation of the findings. Firstly, the number of studies included is small. Only eleven studies on 363 participants were reviewed in this study, and this emphasizes the need for further studies on the topics explored in the context of competitive cycling. In relation to probiotic supplement consumption, only the study carried out by West et al. differentiated between male and female cyclists in relation to a few variables, without exploring any parameters related to cycling performance [46]. Thus, further studies are required on female cyclists, particularly in relation to aerobic and anaerobic performance variables. Moreover, there was a great variation in the type of probiotics, dosage, and duration of supplementation in the studies reviewed. In fact, all studies used a multi-strain probiotic method, which involved different strains and doses in the different protocols, except for the study carried out by West et al. [46]. The authors used a single strain of *Lactobacillus fermentum* [46]. This shows that there is a need to examine the consumption of individual probiotic strains in competitive cyclists so that their effects can be better understood and determined. In addition, the different periods of follow-up were used: from 4 to 16 weeks, with different effects on the competitive cyclists studied. In the study carried out by Pugh et al., a 4-week multi-strain probiotic intake protocol did not influence the cycling performance (time trial) [47]. Based on these results, in the study conducted by Schreiber et al., the 12-week multi-strain protocol did not show any benefit in the cycling tests regarding physiological parameters [48]. On the other hand, the 16-week probiotic intake of multi-strain probiotics led to a significant change in aerobic test parameters, as mentioned in the study conducted by Mazur-Kurach et al. [49]. Nevertheless, the population analyzed, although specific, is still diverse. Amateur cyclists differ significantly from professional cyclists in terms of diet, training volume, training load analysis, and seasonal competitions. All these factors can lead to inconsistency in the overall results and do not allow for a direct comparison between different categories. In addition, for future studies, it would be important to carefully consider the period of administration of probiotic intake, taking into consideration the diet of the athletes as well. None of the studies showed an improvement in anaerobic cycling performance, even in the study that had the longest period of probiotic intake administration. Further studies are needed on this subject. In relation to the composition of gut microbiota, various methods of analysis were used, making it difficult to standardize the results of the studies reviewed. The level of the cyclists also seems to influence the results of the composition of the microbiota. The microbiota of amateur cyclists is different from that of professional cyclists. The level of competitive cyclists should be considered in future studies. Future studies should aim at the composition of the gut microbiota of competitive cyclists, specifically professional cyclists.

## 5. Conclusions

In conclusion, this study sought to determine the characteristics of the gut microbiota in competitive cycling and the impact of probiotics on this particular group of athletes. The current state of knowledge would seem to indicate that competitive cyclists could have a gut microbiota adapted to this sport. Despite the heterogeneity of the studies, the abundance of *Prevotella* would seem to be common to competitive cyclists. However, studies on this topic are still relatively few and show considerable disparities in terms of population (males vs. females, professionals vs. amateurs) and training phase. On the other hand, probiotic supplementation could have an impact on aerobic values and the sensation of physical exertion, as well as on gastrointestinal and respiratory symptoms, intestinal membrane permeability, and anti-inflammatory activity. However, the most significant impact was observed when the duration of supplementation was 16 weeks, using a multi-strain protocol and on a population of amateur cyclists only. If further studies on professional cyclists are conducted, probiotic supplementation could be considered by coaches and trainers.

## Figures and Tables

**Table 1:** Gut Microbiota Composition in Competitive Cyclists.

First Author and Year	Participants	Type of Outcomes/Variables Studied	Main Results
Petersen et al. 2017 [39]	n = 33 competitive cyclists (11 females, 22 males) divided into professional and category 1	Competitive cyclists’ microbiota using whole genome shotgun and metatranscriptomic sequencing	Cyclists clustered into three distinct enterotypes dominated by Prevotella, *Bacteroides*, or a mixed community (*Eubacterium*, *Ruminococcus*, *Akkermansia*). High *Prevotella* abundance (>2.5%) positively correlated with weekly exercise duration and the upregulation of carbohydrate and branched-chain amino acid (BCAA) metabolic pathways. Professional cyclists exhibited higher *M. smithii* transcriptional activity compared to amateurs, with upregulated methane synthesis genes. *M. smithii* presence suggests enhanced gut metabolic efficiency in elite athletes through synergistic cross-feeding mechanisms.
Wiącek et al. 2023 [40]	n = 25 active men (14 amateur cyclists and 11 control group	Assessment of the fecal pH and the abundances of *Bifidobacterium* spp., *Bacteroides* spp., *Akkermansia muciniphila*, and *Faecalibacterium prausnitzii*	Pre-competition gut bacterial abundance and fecal pH remained stable despite variations in diet and endurance. Increased carbohydrate intake during the racing season did not alter these parameters.
Aya et al. 2024 [41]	n = 44 Colombian participants (25 men and 19 women), 16 were weightlifters, 13 were professional road cyclists, and 15 were not athletes.	Two weeks before the national competitions, participants gave fecal samples during their pre-competitive phase. Questionnaire responses were gathered, and GraPhlAn, Pavian, and MicrobiomeAnalyst 2.0 were used to examine the microbial composition and find differences between groups.	Differentially numerous species are revealed by ANCOM-BC2. Road cyclists have higher abundances of Archaea and lower levels of Bacteria. *Planctomycetes*, *Acidobacteria*, and *Proteobacteria* were among the phylum-level variants, although *Bacteroidetes* were the most common. The *Bacteroidaceae*, *Muribaculaceae*, and *Selnomonadaceae* are important families that affect gut microbiota. Weightlifters have distinct relationships with the viral and archaeal communities, whereas cyclists have specialized microbial interactions driven by endurance training.
Fernandez-Sanjurjo et al. 2024 [42]	n = 16 professional cyclists competing in “La Vuelta 2019”	The fecal microbiota populations and SCFA content were analyzed using 16S rRNA sequencing and gas chromatography, respectively.	Strong predictive value for *Bifidobacteriaceae*, Coriobacteriaceae, *Erysipelotrichaceae*, and Sutterellaceae dynamics (r = 0.83 for ranking; r = 0.81 for accumulated time). Positive correlations between Coriobacteriaceae and acetate (r = 0.530)/isovalerate (r = 0.664), and *Bifidobacteriaceae* with isobutyrate (r = 0.682). No correlation between SCFA levels and performance. Pre-competition complex carbohydrate intake was positively linked to baseline *Erysipelotrichaceae* (r = 0.956); intra-competition simple carbohydrate supplementation negatively impacted *Bifidobacteriaceae* (r = −0.650). Ecological modeling outperformed single-taxon analysis in characterizing microbiota-performance links.
Ampe et al. 2025 [43]	n = 27 (14 elite female cyclists and 13 non-athlete female controls)	16S rRNA gene sequencing and SCFA quantification of fecal samples collected during the off-season (reduced training period).	Significant enrichment of *Bacteroidota* and reduction in *Firmicutes* in cyclists vs. controls. Lower microbial diversity in cyclists (Shannon index, *p* < 0.05). Depletion of fiber-degrading families (*Lachnospiraceae*, *Ruminococcaceae*) associated with high-carbohydrate/low-fiber intake. Fecal SCFA concentrations remained stable, suggesting functional compensation.
Shalmon et al. 2024 [44]	n = 58: 18 amateur cyclists (9 males), 22 runners (13 males), and 18 control subjects (9 males)	Fecal samples were analyzed using 16S rRNA sequencing to characterize species composition, and both alpha and beta diversity metrics were used to evaluate differences between groups. Participants also underwent a VO_2_max test and a time-to-exhaustion trial performed at 85% of their VO_2_max, during which blood lactate was sampled every five minutes.	Alpha diversity differed significantly between cyclists and runners (*p*-adj < 0.001), with male cyclists exhibiting markedly lower diversity than male runners (*p*-adj < 0.001). Comparative taxonomic profiling across cyclists, runners, and controls revealed shifts in the abundance of fifteen bacterial taxa. Several microbial features were linked to performance metrics: six taxa in cyclists and eight in runners showed significant positive correlations with training volume, time-to-exhaustion, VO_2_max, or blood lactate concentrations.
Aya 2025 [45]	n = 29 Colombian athletes: 16 elite weightlifters and 13 professional cyclists	Fecal and plasma samples obtained one month prior to the international competition were analyzed using metagenomic, metabolomic, and lipidomic approaches.	Key metabolic pathways—including those related to aromatic amino acid synthesis, arginine production, and folate metabolism—were enriched in both athlete groups. Plasma metabolomic and lipidomic data showed clear distinctions between the groups. The results emphasize the interplay between gut microbiota features and systemic metabolic responses shaped by sport-specific requirements.

**Table 2:** Probiotics Supplements in Competitive Cyclists.

**First Author and Year**	**Participants**	**Probiotic Dose Intake/** **Intervention Length**	**Type of Outcomes/Variables Studied**	**Main Results**
West et al. 2011 [46]	n = 99 amateur cyclists (64 M and 35 F) divided into: - Probiotic group n = 47 (29 M; 35.2 ± 10.3 years and 18 F; 36.5 ± 8.6 years) - Placebo group n = 50 (33 M; 36.4 ± 8.9 years and 17 F; 35.6 ± 10.2 years)	1 × 10^9^ Probiotic supplement: *Lactobacillus fermentum* (PCC) per day for 11 weeks	- The self-reported number, duration, severity and combined load of gastrointestinal symptoms illness (GI). - The self-reported number, duration, severity and combined load of upper respiratory tract symptoms illness (URTI) and lower respiratory illness. - Stool *Lactobacillus fermentum* counts. - Serum cytokine marker analysis: interleukin (IL)-1RA, IL-6, IL-8, IL-10, tumor necrosis factor (TNF)-a and interferon gamma (INF-g).	Gastrointestinal assessments revealed a two-fold increase in the incidence and duration of mild symptoms across both sexes; however, probiotic-supplemented males reported a severity score 0.7 points lower than the placebo group, an effect that intensified alongside higher training loads. Regarding respiratory health, while the impact on URTIs remained inconclusive, probiotic use was associated with a roughly 50% reduction in the number, duration, and severity of lower respiratory symptoms in males. Conversely, female cyclists experienced a two-fold increase in the frequency of lower respiratory symptoms, although their overall severity was reduced. These clinical outcomes were mirrored by shifts in microbiota composition, where total Lactobacillus counts saw a moderate 330% increase in the male probiotic group compared to a 44% decrease in the placebo group, while differences among females remained minimal.
Pugh et al. 2020 [47]	n = 7 trained cyclists (M); 23 ± 4 years divided into Probiotic group or Placebo group	1 × 10^10^ multi-strain Pro (commercially available probiotic). The Probiotic supplement contained the active strains: *Lactobacillus acidophilus* (CUL60), *Lactobacillus acidophilus* (CUL21), *Bifidobacterium bifidum* (CUL20), and *Bifidobacterium* animalis subsp. lactis (CUL34) per day for 4 weeks, separated by a 2-week washout period	- Lipid and carbohydrate oxidation after an oral dose of maltodextrin during a trial of 120 min of cycling at 55% maximal aerobic power output, followed by a 100 kJ time trial. - Blood parameters: Plasma glucose, lactate, nonesterified fatty acids (NEFAs), and glycerol. - Gastrointestinal permeability: lactulose and rhamnose ratio (LR) through blood samples. - Subjective symptoms of discomfort	Probiotic supplementation significantly influenced metabolic substrate utilization during exercise, characterized by slightly higher peak oxidation rates of ingested maltodextrin (*p* = 0.016) and increased mean carbohydrate oxidation (*p* = 0.038), alongside a mild decrease in fat oxidation (*p* = 0.021). These metabolic shifts were supported by small but significant increases in glucose absorption, plasma glucose, and insulin concentrations, coupled with a reduction in non-esterified fatty acids and glycerol within the probiotic group. Despite these physiological changes, the intervention yielded no significant impact on time-trial performance, gastrointestinal damage, or intestinal permeability (*p* > 0.05).
Schreiber et al. 2021 [48]	n = 27 (M); ranked elite or category 1 level competitions divided into: - Experimental group n = 11; 25.9 ± 4.6 years - Control group n = 16; 29.5 ± 6.2 years	1 × 10^10^ multi-strain Probiotic supplement with five strains: at least (≥) 4.3 × 10^9^ CFU *Lactobacillus helveticus* Lafti L10 (28.6%), ≥4.3 × 10^9^ CFU *Bifidobacterium animalis* ssp. lactis Lafti B94 (28.6%), ≥3.9 × 10^9^ CFU Enterococcus faecium R0026 (25.7%), ≥2.1 × 10^9^ CFU *Bifidobacterium* longum R0175 (14.3%) and ≥0.4 × 10^9^ CFU *Bacillus subtilis* R0179 (2.8%) per day for 12 weeks	- Personal and GI symptoms questionnaire. - VO_2_max test. - Time to fatigue (TTF) test. - Inflammatory Blood markers analysis: IL-6, TNFα and CRP	A lower incidence of GI symptoms during training sessions in the experimental group compared to the control group was registered (*p* = 0.04). Especially the incidence of nausea (*p* = 0.01), belching (*p* = 0.04), and vomiting (*p* = 0.04). No significant effects in the VO_2_max test and TTF test in terms of physiological variables were found. Only RPE (rate of perceived exertion) scores during the TTF test were found to have changed between groups (*p* = 0.04). Not all inflammatory markers were significantly changed.
Mazur-Kurach et al. 2022 [49]	n = 26 (M) amateur road cyclists assigned to: - Probiotic group n = 13; 23.25 years - Control group n = 13; 21.28 years	1 × 10^11^ multi-strain Probiotic supplement, which contained: *Lactobacillus plantarum*, *Lactobacillus casei*, *Lactobacillus rhamnosus*, *Bifidobacterium breve*, *Lactobacillus acidophilus*, *Bifidobacterium longum*, *Bifidobacterium bifidum*, *Bifidobacterium infantis*, *Lactobacillus helveticus*, *Lactobacillus fermentum*, *Lactobacillus bulgaricus*, *Lactococcus lactis*, and *Streptococcus thermophilus* per day for 16 weeks. The follow-up measurements were the baseline, after 4 weeks, after 12 weeks, and the final measurement after 16 weeks.	- Aerobic Performance (Incremental Test): Maximal oxygen uptake (VO_2_max), exercise duration, maximum power (Pmax), maximal heart rate (HRmax), and perceived exertion (Borg-20 scale). - Anaerobic test (Modified Wingate Test): Maximal anaerobic power, level of total work, average power per revolution, mean time to achieve maximal anaerobic power and time to maintain maximal anaerobic power. - Anthropometry: Body mass (BM), fat mass (FM), and lean body mass (LBM) - Blood lactate levels in Aerobic and Anaerobic Tests: Pre-exercise, after 3 min, 10 min and 20 min of exercise - Blood Biochemistry: Serial monitoring (pre-exercise, 3, 10, and 20 min post) of lactate levels, inflammatory cytokines (IL-1β, IL-6, IL-8, IL-10, TNF-α), IgA, and oxidative stress markers (TOS, TAS). **-** Intestinal Permeability: Fecal concentration of zonulin and α1-atitrypsin	Following 16 weeks of probiotic supplementation, significant improvements were observed in aerobic performance, characterized by increased VO_2_max and time to exhaustion, alongside a reduction in maximal heart rate (*p* < 0.05). Metabolic recovery was enhanced, with blood lactate concentrations post-aerobic testing significantly lower at 10 and 20 min compared to baseline (*p* < 0.01); notably, the probiotic group also reported lower perceived physical fatigue (*p* = 0.0026). While anaerobic capacity remained unaffected, a slight but significant increase in muscle mass was noted (*p* = 0.02). Furthermore, the intervention strengthened intestinal barrier function, evidenced by reduced zonulin levels (*p* = 0.0035), and bolstered mucosal immunity via increased IgA (*p* = 0.01). These changes were accompanied by a systemic reduction in inflammatory and oxidative stress markers, including TNF-alpha, IL-6, IL-10, and TOS (*p* < 0.01).

## Data Availability

No datasets were generated or analyzed during the current study.

## Supplementary Materials

The following supporting information can be downloaded at https://www.mdpi.com/article/10.3390/nu18060991/s1. Table S1: Full Search Strategy.
