# Supplementary material for: Gut Microbiota Profile and the Impact of Probiotic Supplementation in Competitive Cyclists: A Scoping Review
Source: Nutrients. 2026 Mar 20;18(6):991. doi: 10.3390/nu18060991 (PMC13029010; doi:10.3390/nu18060991)
Supplement: Supplementary file 1 [file nutrients-18-00991-s001.zip › nutrients-4189494-supplementary.pdf]

Supplementary Material Table S1. Full Search Strategy.

**PCC**
**Population** Well-trained competitive cyclists from any discipline: road cycling, mountain biking, track cycling.
**Concept** The composition of the gut microbiota of cyclists and probiotic intake.
**Context** The research context is limited to competitive endurance environments, focusing on the dual outcomes of athletic performance (e.g., power output, time-trial results) and gastrointestinal integrity. This includes the assessment of gut microbiota modulation as a strategy to mitigate exercise-induced gastrointestinal distress and its subsequent impact on the athlete's systemic health and training consistency.
**Limitations** Peer-review articles, published up to the 1st of November 2025. Only Randomized Controlled Trials (RCTs) and Randomized Crossover Trials were considered for probiotic intake. No studies evaluating endurance athletes in general, or where it is not specified that they are cyclists. No studies on recreational cyclists or where it was not possible to identify the difference from competitive cyclists, no studies published in languages other than English, no reviews, systematic reviews and meta-analysis.

Keywords were arranged into two subsets.
Subset A comprised the keywords: “gut microbiota”, “microbiome”, “competitive cyclists”, “elite cyclists”, ”professional cyclists”, and “amateur cyclists”.
Subset B comprised the keywords: “probiotic supplementation”, “probiotics”, “cycling”, “competitive cycling”, “elite cycling”, “professional cycling” and “amateur cycling”.
Within each subset the words were combined with the Boolean operator OR. Each subset was combined with the Boolean operator AND.

Search array: (“gut microbiota” OR “microbiome”) AND (“competitive cyclists” OR “elite cyclists” OR “professional cyclists” OR “amateur cyclists”), (“probiotic supplementation” OR “probiotics”) AND (“cycling” OR “competitive cycling” OR “elite cycling” OR “professional cycling” OR “amateur cycling”)
